# Supplementary figures and images for: Discovery and Characterization of Actively Replicating DNA and Retro-Transcribing Viruses in Lower Vertebrate Hosts Based on RNA Sequencing
Source: Viruses. 2021 May 31;13(6):1042. doi: 10.3390/v13061042 (PMC8227577; doi:10.3390/v13061042)

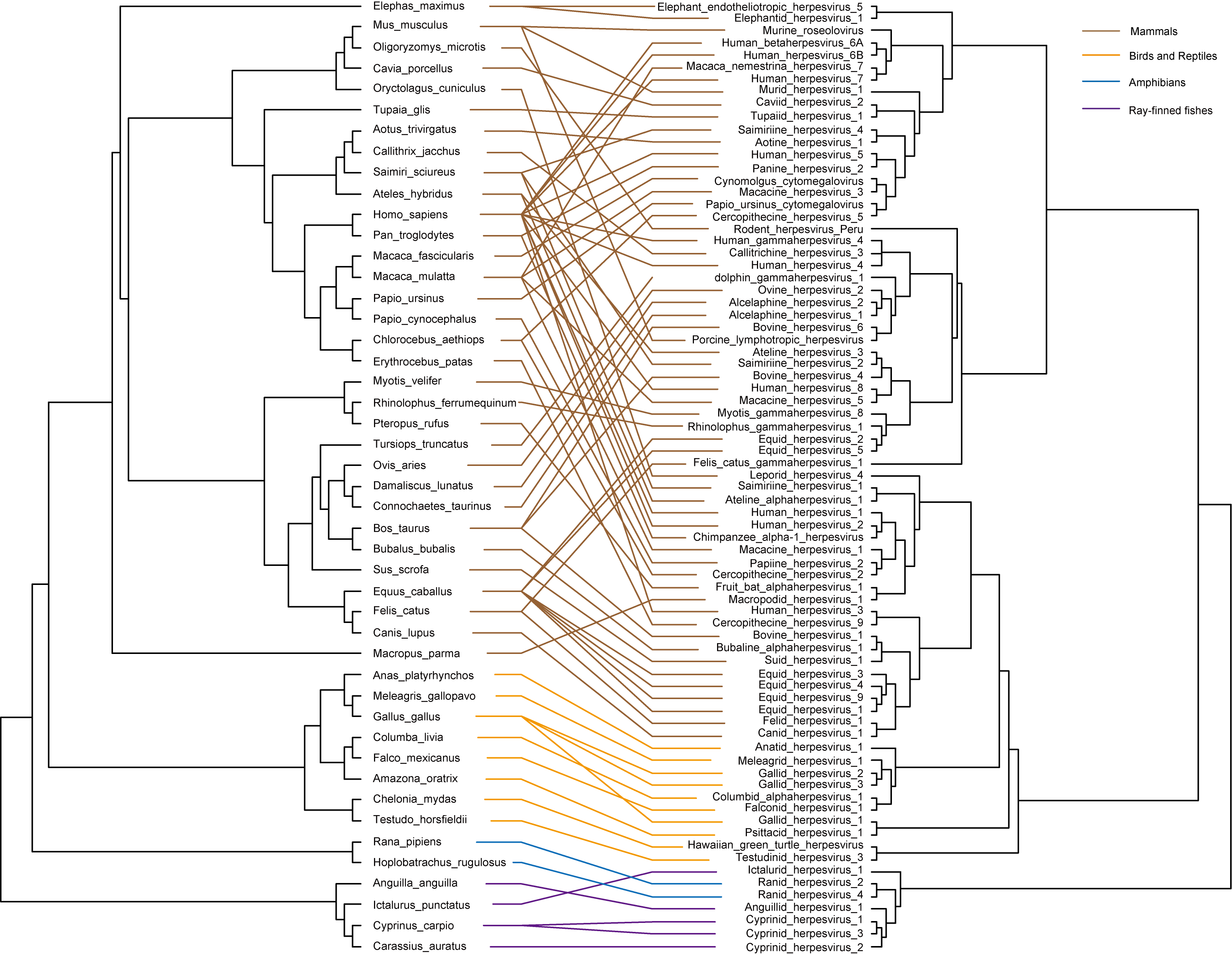

Supplement: Supplementary file 1 [file viruses-13-01042-s001.zip › Figure_s1.tif]

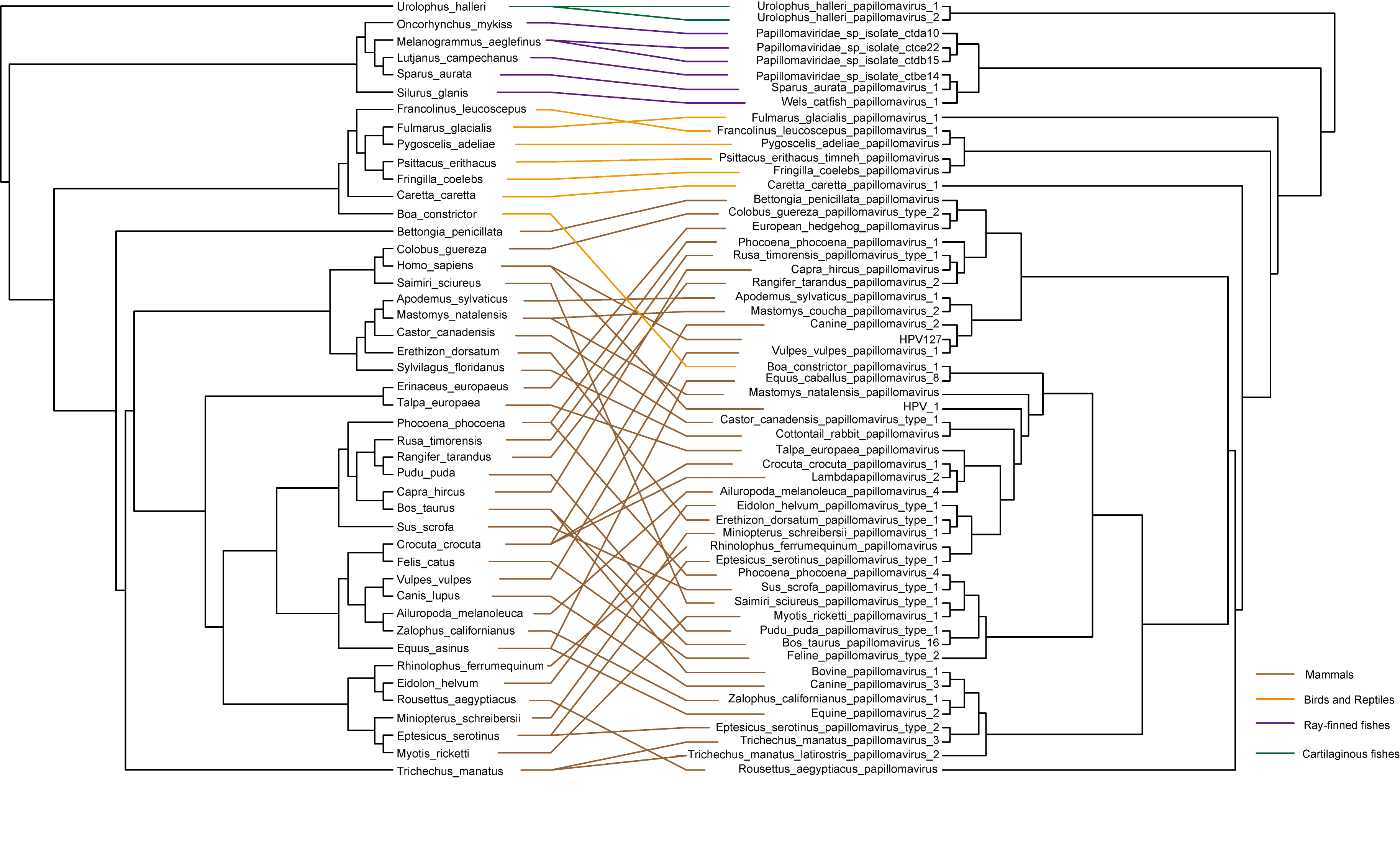

Supplement: Supplementary file 1 [file viruses-13-01042-s001.zip › Figure_s2.tif]

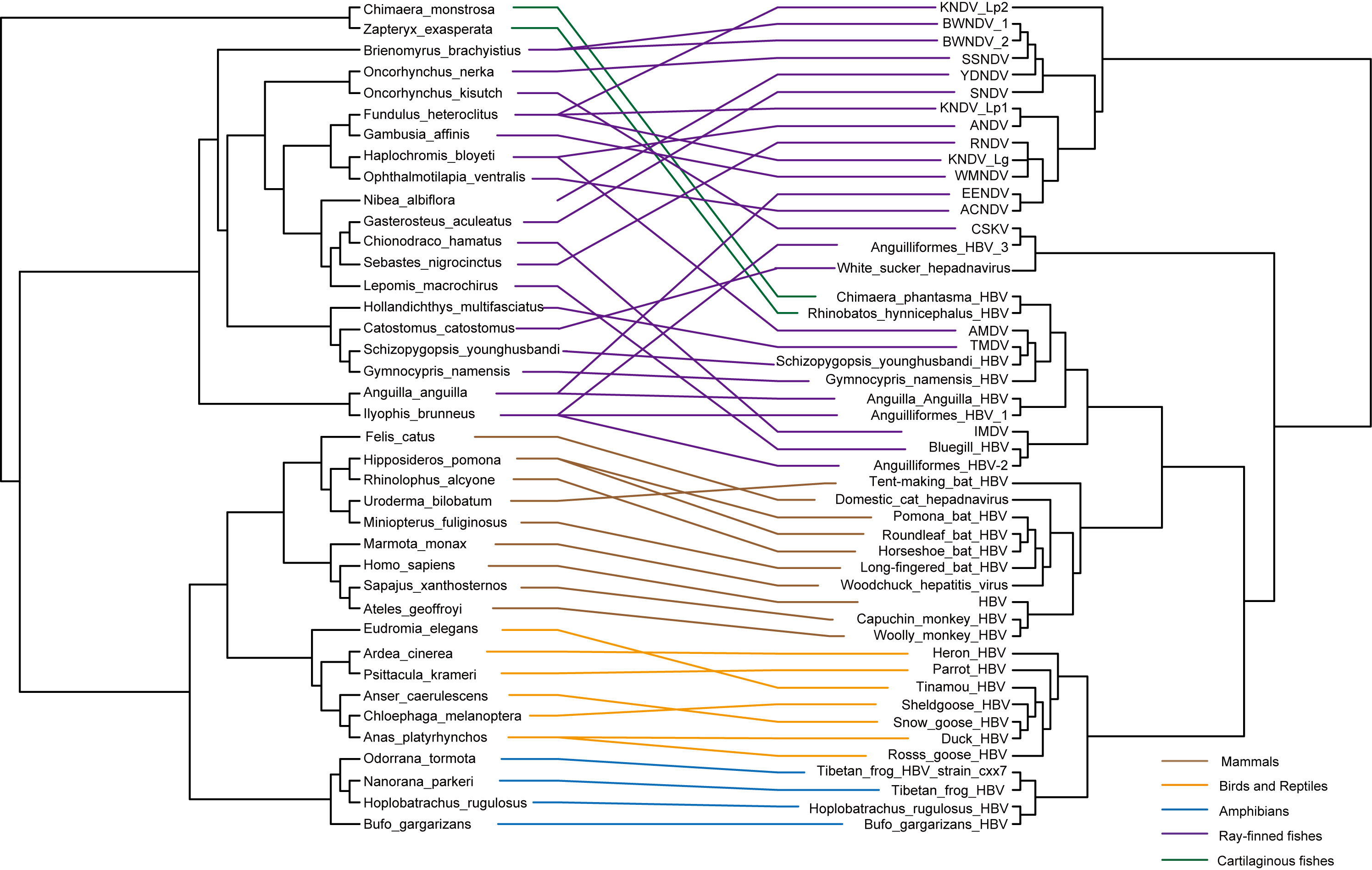

Supplement: Supplementary file 1 [file viruses-13-01042-s001.zip › Figure_s3.tif]

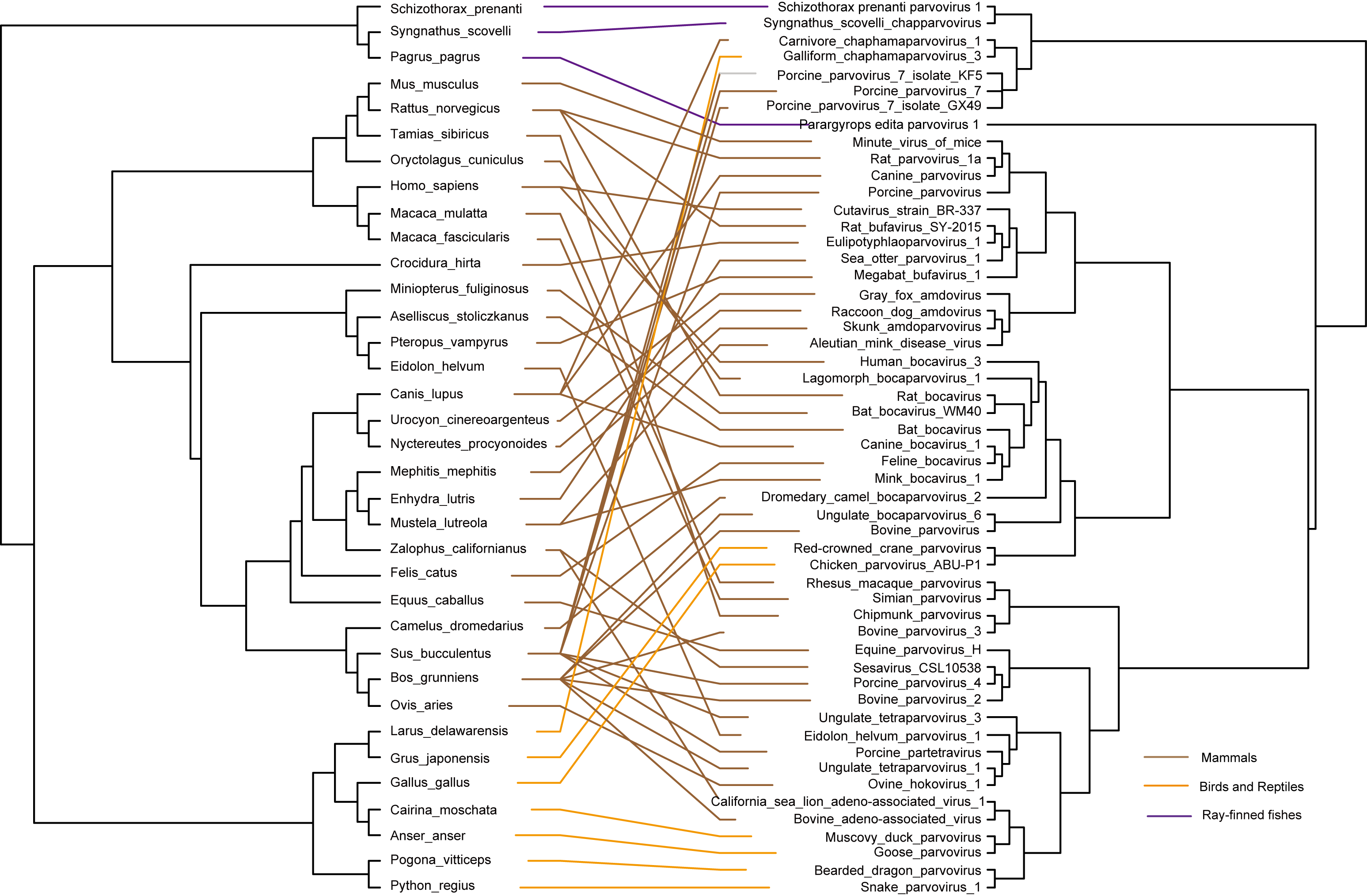

Supplement: Supplementary file 1 [file viruses-13-01042-s001.zip › Figure_s4.tif]
